# Diabetes Comorbidity and Quality of Life in Patients with Cancer: A Prospective Study in an Integrative Oncology Setting

**DOI:** 10.3390/jcm14061800

**Published:** 2025-03-07

**Authors:** Sameer Kassem, Noah Samuels, Orit Gressel, Nili Stein, Miri Golan, Eran Ben-Arye

**Affiliations:** 1 Faculty of Medicine, Technion-Israel Institute of Technology, Haifa 3436212, Israel; oritgresselraz@gmail.com (O.G.); eranben@netvision.net.il (E.B.-A.); 2 Department of Medicine, Carmel Medical Center, Clalit Health Services, Haifa 3436212, Israel; 3 Center for Integrative Complementary Medicine, Shaare Zedek Medical Center, Faculty of Medicine, Hebrew University of Jerusalem, Jerusalem 9103102, Israel; noahs@szmc.org.il; 4 Integrative Oncology Program, The Oncology Service, Lin, Carmel, and Zebulun Medical Centers, Clalit Health Services, Haifa 3515210, Israel; mirigo1@clalit.org.il; 5 Department of Community Medicine and Epidemiology, Carmel Medical Center, Haifa 3436212, Israel; stein_nili@clalit.org.il

**Keywords:** integrative oncology, diabetes mellitus, cancer, pain, quality of life

## Abstract

**Background:** Research on quality of life (QoL)-related concerns among patients with both diabetes mellitus (DM) and cancer is limited. This study compared the QoL-related concerns and characteristics among chemotherapy-treated patients with cancer and DM to those without DM. **Methods:** Chemotherapy-treated patients were evaluated during integrative oncology (IO) consultations, which included evidence-based complementary therapies recommended by their healthcare providers to address quality of life (QoL) concerns. During these consultations, the participants were assessed for comorbidities, including diabetes mellitus (DM). QoL-related concerns were measured using the Edmonton Symptom Assessment Scale (ESAS) and the European Organization for Research and Treatment of Cancer Quality of Life Questionnaire (EORTC QLQ-C30). **Results:** Of the 1171 patients referred for an IO consultation, 272 (23.2%) had an established diagnosis of DM. The DM patients were older, presented with more advanced stages of cancer, and had more chronic comorbidities (*p* < 0.001). While fatigue was the most frequently reported QoL-related concern in both groups, the patients with DM had more severe pain scores in the ESAS (4.9 vs. 4.4, *p* = 0.022) and lower ESAS well-being scores (5.9 vs. 5.5, *p* = 0.021). **Conclusions:** Chemotherapy-treated patients with cancer and DM are characterized by higher rates of comorbidities and report more severe scores for pain and for poorer general well-being. Oncologists and diabetologists should consider referring patients with both diagnoses for an IO consultation to address their QoL-related concerns. More research is needed to understand the impact of IO consultations and treatments on well-being among patients diagnosed with both DM and cancer.

## 1. Introduction

Type 2 diabetes mellitus (DM) has long been associated with an increased risk of the development of cancer, with poorer treatment outcomes [1] and an increased risk of long-term overall mortality [2]. DM and cancer share many risk factors [3], with anti-cancer treatments often being associated with impaired glycemic control or new-onset DM [4]. Patients with breast cancer and DM have been shown to present with more advanced stages of disease, requiring more aggressive anti-cancer treatment regimens [5]. In addition, a diagnosis of DM has been associated with a lower likelihood that the patient will receive guideline-concordant chemotherapy [6]. A 2016 systematic review found that patients with cancer and DM report a worse health-related quality of life (QoL) and functioning than those without DM [7]. Despite the higher rates of reported symptoms such as fatigue, depression, and anxiety among patients with DM, little research has been published on the implications of DM regarding patients’ ability to overcome chemotherapy-related QoL-related concerns. In this context, Mailliez et al. found that oncology patients with DM had a significantly higher incidence of severe adverse events within 90 days of the initiation of chemotherapy [8]. In a study by Kleckner et al., DM was associated with more severe cancer-related fatigue among patients undergoing chemotherapy for breast cancer [9]. Moreover, patients with cancer who have more prolonged and uncontrolled DM have been shown to report a greater impairment in emotional functioning and global health status [10]. Chemotherapy-treated patients with non-metastatic breast cancer and DM had more emergency room and inpatient admissions and higher rates of infection and required more frequent changes in their oncology treatment regimens [11]. In their study of a cohort of patients with resectable pancreatic cancer and DM, Kuo et al. found a correlation between the duration of DM and self-care, as well as patient-reported QoL-related outcome measures [12]. In a more recent study, Ose et al. found that patients with cancer and DM reported significantly worse scores for anxiety, depression, fatigue, pain interference, and physical function when compared to those without DM [13].

The advances made in the treatment of cancer, with an increase in survival outcomes and life expectancy, highlight the need to manage chronic comorbidities, including DM. Cancer survivors are more likely to have hypertension and DM, both of which can significantly increase overall morbidity and mortality [14]. The American Heart Association (AHA) has emphasized the importance of cardio-oncology rehabilitation to address cardiovascular outcomes in cancer patients and survivors, highlighting the need for a multimodal approach to improve cardiovascular health in this patient population [15].

Many of today’s leading cancer centers provide integrative oncology (IO) therapies as part of their supportive and palliative care services [16]. The Society for Integrative Oncology (SIO) and the American Society of Clinical Oncology (ASCO) have co-published several clinical practice guidelines that address cancer-related pain [17]; anxiety and depression [18]; and cancer-related fatigue [19]. Observational pragmatic research supports the effectiveness of IO programs for the treatment of chemotherapy-related toxicities (e.g., chemotherapy-induced peripheral neuropathy) [20] and QoL-related concerns, including pain [21]. However, to the best of our knowledge, no evidence-based IO practice guidelines have yet been published on the treatment of patients dually diagnosed with cancer and DM.

The present study examined the QoL-related concerns among chemotherapy-treated patients enrolled in the IO program, comparing those with a diagnosis of DM to those without this comorbidity.

## 2. Materials and Methods

This study was designed within a pragmatic, prospective, patient-preference, controlled, and non-randomized research methodology. Patients with cancer were recruited between May 2013 and October 2023 from the Lin and Zebulon Medical Centers, both part of the Oncology Service of the Clalit Healthcare Services in Haifa, Israel. These two community-based medical centers offer a comprehensive array of IO modalities which are provided in their ambulatory oncology services, with a specific focus on QoL-related concerns. All patients undergoing active anti-cancer treatment at these centers are eligible for IO consultations and treatments, without charge. The study hypothesis was that the QoL-related concerns among patients with DM would be more severe than in those without DM.

Eligibility for study inclusion was based on the following criteria: an age of 18 years or older and undergoing adjuvant, neo-adjuvant, or palliative chemotherapy regimens. Patients were referred to an initial IO consultation by their oncology healthcare providers (HCPs) at either of the participating study centers. Referring HCPs include a multidisciplinary team which comprises oncologists, oncology nurses, psycho-oncologists, oncology surgeons, and specialists in palliative care. IO consultations are conducted by integrative physicians (IPs) affiliated with the study centers, who are MDs trained in providing comprehensive supportive care which is tailored to the QoL-related concerns commonly reported by cancer patients.

Demographic and clinical data were elicited from patient interviews, as well as from the patients’ electronic medical records. This included variables such as age, gender, primary language spoken, socioeconomic status, and chronic comorbid conditions. A Charlson Comorbidity Index score was derived from the information gleaned from the electronic medical records. Patients with cancer who also had a diagnosis of DM were identified through direct questioning, as well as an ICD-9 code appearing in their electronic medical records.

The IP consultation explores QoL-related concerns and issues related to well-being; health-belief models of care; and the past/present/planned use of complementary medicine. Expectations from the consultation are addressed, and a comprehensive IO evaluation is conducted, addressing emotional concerns (anxiety, depression, and insomnia); gastrointestinal-related symptoms; fatigue; pain; and daily functioning.

The severity of QoL-related concerns was evaluated using the Edmonton Symptom Assessment Scale (ESAS), asking the respondent to score the severity of their symptoms from the previous 24 h period (from 0, none, to 10, the worst possible severity), and the European Organization for Research and Treatment of Cancer Quality of Life (EORTC QLQ-C30) tool, which scores symptoms and overall health from the past 7 days. EORTC items are scored both individually and in calculated QoL-related clusters from 1 (the least) to 4 (the most severe), as well as calculating an integrated score which ranges from 0 (the worst) to 100 (the least severe).

Statistical analyses were performed using IBM SPSS Statistics version 28.0 (IBM, New York, NY, USA). Continuous variables were presented using means and standard deviations (SDs) or medians and interquartile ranges (IQRs), while categorical variables were represented as counts and proportions. Comparisons of the patient characteristics between patients with cancer and DM and those without DM were performed using a Chi-square test for categorical variables. For continuous variables, either an independent *t*-test or the Mann–Whitney U test was used, depending on the data distribution. For the multivariate analysis, logistic regression was used to analyze the association between DM and high ESAS scores (ESAS score > 5 for pain, fatigue, and well-being), adjusted for demographic and clinical characteristics including age, gender, country of birth, socioeconomic status, cancer type, metastatic disease, oncology treatment setting (adjuvant/palliative), Charlson Comorbidity Index scores, and cancer-related complementary medicine use. Odds ratios (ORs) with 95% confidence interval (CIs) are presented. A two-tailed significance level of 0.05 was used for all of the statistical tests in this study. Since this was an exploratory and descriptive study, with all patients enrolled in the IO program being eligible for study inclusion, no sample size estimation or power calculations were performed.

## 3. Results

The patient characteristics are presented in Table 1. Of the 1171 patients with cancer referred to and that attended an IP consultation, 272 (23.2%) had a co-diagnosis of DM. The patients with DM were more likely to be older, to be male (vs. female), and to not have been born in Israel; less likely to have a higher socioeconomic status and to have been diagnosed with breast cancer; and more likely to have gastrointestinal, lung, or urinary tract cancer and to have presented with metastatic disease and to be undergoing chemotherapy in a palliative care setting. The DM group had more comorbidities, which included hypertension, hyperlipidemia, obesity, ischemic heart disease, chronic renal failure, and stroke, and had significantly higher mean Charlson Comorbidity Index scores.

The patients’ stated treatment goals are presented in Table 2. When analyzing the entire cohort, most of the patients (with or without DM) identified management of their fatigue as the primary goal of IO treatment, followed by gastrointestinal symptoms, emotional concerns, and pain. No significant differences were observed between the patients with DM and those without. However, in the breast cancer patients, the DM group was less likely to report emotional-related concerns than the non-DM group. Among the patients with gynecological cancer, patients with DM were more likely to report emotional and gastrointestinal concerns than those in the non-DM group.

The assessments of QoL-related concerns in the cohort are presented in Table 3. Patients with DM reported significantly worse pain and well-being scores in the ESAS than those without DM. Conversely, the patients with DM had less severe nausea scores. No significant differences were observed between the two groups for the other QoL-related concerns. However, in a subgroup analysis, the DM patients with gynecological cancer reported significantly more severe pain, with borderline significance for fatigue severity using both the ESAS and EORTC QLQ-C30 tools.

The findings of the multivariate analysis testing for an association between DM and high ESAS scores (ESAS score > 5) for pain, fatigue, and well-being are presented in Table 4. DM was significantly associated with a greater likelihood of high ESAS scores for pain among the entire cohort (OR: 1.47; CI: (1.02–2.12); *p* = 0.038). Among the most frequent cancer types, this association remained significant in the patients with gynecological cancers (OR: 1.77; CI: (1.24–2.52); *p* = 0.002).

## 4. Discussion

The present pragmatic study compared patients with cancer who had a co-diagnosis of DM with those without. The patients in the DM group tended to be older and have more comorbidities than those without DM. At the same time, the QoL-related concerns were, for the most part, of a similar severity in both groups of patients, though the ESAS scores for pain were significantly more severe in the DM group.

The baseline differences between the two groups of patients should be interpreted within the context of the well-established characteristics of patients with DM, a condition which is more commonly found among older adults. This may account for the relatively older age of the DM group in this study [22]. The high proportion of females in this cohort (in both the DM and non-DM groups) may be attributed, at least in part, to a possible referral bias, reflecting the ongoing collaboration between the IO service and the breast and gynecologic oncology departments at the two participating study centers. The higher proportion of non-native Israelis in the DM group is in keeping with a previously reported study which found an association between immigrant status and an increased prevalence of DM. This correlation has been attributed to factors such as a change in lifestyle after migration, with immigrant patients adopting a more sedentary level in their physical activity and changes in dietary habits. In addition, increased emotional stress due to acculturation, socioeconomic challenges, limited access to healthcare, and language barriers have all been shown to contribute to the development of DM in immigrant populations as well [23].

In addition to the above, an increased prevalence of DM has been found among patients from a lower socioeconomic status (SES), with lower rates of DM among higher-SES populations [24]. This may be explained by a number of key factors, such as the fact that patients from higher-SES populations tend to exhibit greater health awareness, which in turn can lead to better management of diabetes risk factors and the adoption of prevention strategies. These patients often have greater access to nutritious food options, enabling them to make healthier dietary choices that can mitigate the risk of developing DM. Moreover, higher-SES populations are more likely to engage in regular physical activity due to better access to recreational facilities and safer environments for exercise. Financial stability and robust social support can also contribute to lower stress levels in this population, which positively impact overall health and reduce the risk of developing chronic conditions such as DM. These factors collectively underscore the significant influence of socioeconomic status on the prevalence of DM, with implications for cancer care and IO programs treating this subpopulation of patients.

In the present study, the patients with DM attending an IP consultation were more likely to present with an advanced stage of cancer, with a greater incidence of metastatic disease, and to be undergoing treatment in the setting of palliative care. These findings may all be partially attributed to the older age and more advanced disease upon presentation found in the DM groups. Breast cancer was found to be less prevalent in this group as well, despite the established association between this form of cancer and DM [25]. In contrast, patients with DM were more likely to be diagnosed with gastrointestinal and lung cancer. These findings may have been associated with a referral bias among the HCPs at the study centers or else with the patients’ willingness to attend the freely provided IO consultation and treatments and not necessarily with the comorbidity of DM.

The significantly greater incidence of chronic multi-morbidity observed among the patients in the DM group is consistent with the findings of other research on this patient population. Chronic conditions such as hypertension, hyperlipidemia, and obesity are integral components of metabolic syndrome, which is frequently linked with insulin resistance and DM [26]. It is thus to be expected that other comorbidities of DM and metabolic syndrome would include ischemic heart disease, nephropathy, and cerebrovascular disease. These and other age-associated comorbidities contributed to the higher Charlson Comorbidity Index scores found in the DM group.

Peripheral neuropathy is a well-known pain-related complication of both DM [27] and the use of many anti-cancer drugs (e.g., platinum- and taxane-based drugs) [28]. As such, it would be expected that chemotherapy-treated patients with DM would be more likely to list pain as a major concern, a finding that was not found in the present cohort. This may reflect the high frequency of reported pain among all patients with cancer, as well as the timing of the referral by the HCP to an IP consultation prior to the initiation of chemotherapy. At the same time, the ESAS scores indicated a significantly higher frequency of reported pain and worse general well-being in the DM group, with borderline significance in the EORTC scores. These findings align with those of the study by Trendowski et al., which identified DM comorbidity as a significant risk factor for chemotherapy-induced peripheral neuropathy among African American cancer survivors [29]. However, our study focused on patients undergoing active chemotherapy treatments, many of them with advanced cancer and in a palliative care setting. In addition, the varied ethnic and cultural backgrounds of our cohort limit the ability to draw direct comparisons between these two studies.

The EORTC pain scores were significantly more severe among patients with breast and gynecological cancers in the DM group. These findings are in keeping with a recently published study which showed poorer patient-reported outcomes, including increased pain, in cancer patients with DM comorbidity [13]. In a meta-analysis conducted by Xie et al., DM was identified as a significant risk factor for developing acute radiation dermatitis following radiotherapy among breast cancer patients [30]. In the present study, the adverse effects of radiation treatment in breast cancer patients were not examined, including radiation-related symptoms. The perception of pain among patients with cancer and DM needs to be better understood within the context of dysesthesia, anticipated pain/disability, and the impact on daily functioning while relating it to prior experience with symptoms of diabetic neuropathy. The findings of the present study emphasize the importance of routinely questioning cancer patients about their pain and general well-being, especially if they have a diagnosis of DM, while they have been found to be less likely to list this as an important QoL-related concern.

The findings of the present study highlight the need for diabetes physicians and oncologists, as well as integrative physicians who are co-designing the IO treatment goals, to be aware of the increased risk of the development of neuropathic pain and other symptoms among patients with both cancer and DM. The current clinical guidelines, such as those published by the SIO and ASCO on IO therapies for cancer-related pain, do not address DM as a relevant co-morbidity [17]. Further research is needed to characterize patients with cancer and DM better, adopting a more focused and directed DM-centered approach tailored to this unique patient population. The referral bias identified needs to be considered among the barriers to the accessibility of the freely provided IO service, including diversity, equity, and inclusion (DEI)-related factors [31]. These include the patient’s age, primary language, gender, and expression of emotional and spiritual-related concerns.

The present study has several limitations, mostly as a result of the pragmatic and non-randomized methodology, which introduces an inherent selection bias. Patients who choose to attend the IP consultation and IO treatments may have a health-belief model with an affinity towards a healthier lifestyle or resilience when compared to these qualities in the general population of cancer patients undergoing chemotherapy. Although standardized referral criteria have been established, awareness and understanding of these criteria among the referring HCPs may vary, with a potential for referral and selection bias. Furthermore, this study was conducted in two centers in northern Israel, both of which share diverse intercultural characteristics. Notably, in contrast to these centers, not all oncology centers in Israel offer IO care as an integral part of their supportive and palliative care services.

The geographical and cultural context of these study centers may further limit the generalizability of our findings. Conducting a multicenter, international, and prospective study would greatly enhance the value and applicability of the research goals to other centers, both in Israel and globally. In addition, although this study used validated and widely accepted assessment tools (the ESAS and EORTC QLQ-C30), these were subjectively scored by the study participants. As such, the symptom scores may have been influenced by individual beliefs and perceptions, which can vary among patients and do not necessarily reflect the comorbidity with DM. Assessing QoL-related concerns is complex and requires caution when interpreting the results. The differences in the baseline scores between the two study groups, including the older age and more advanced cancer in those with DM, further precludes us reaching conclusions and ascertaining causality. It is important to note that patients in the DM group had been referred by their oncology HCPs to an IP consultation without addressing factors related to their DM or other comorbidities. A number of confounding factors associated with DM-related comorbidities were not addressed. In addition, the lack of adjustments for multiple comparisons and a long-term follow-up may have adversely affected the robustness of the data presented. Nevertheless, the study findings highlight the need for further examination of this subset of chemotherapy-treated patients with both cancer and DM, as well as other related comorbidities.

## 5. Conclusions

The present study emphasizes the need to actively and systematically assess pain and pain-related symptoms in cancer patients, particularly those with a comorbidity of DM. Patients with both comorbidities were found to experience greater pain and poorer overall well-being than those without DM, though they did not identify these as QoL-related concerns. Further research is needed to understand this subgroup of cancer patients better and to develop new diagnostic and therapeutic strategies aimed at improving the QoL among cancer patients in general and among those with a comorbidity of DM in particular.

## Figures and Tables

**Table 1:** Characteristics of patients at an initial integrative oncology consultation: patients with diabetes mellitus (DM) vs. those without DM.

Characteristic	DM n = 272	Non-DM n = 899	*p* Values
**Age** Mean ± SD (median)	67.1 ± 9.1	58.9 ± 13.2	<0.001
**Gender/sex** Female	184 (67.6)	740 (82.3)	<0.001
**Primary language** Hebrew	195 (71.7)	596 (66.3)	0.096
**Country of birth** Israeli-born	141 (51.8)	544 (60.5)	0.011
**Residence** Haifa vs. suburbs and periphery	193 (71.0)	597 (66.4)	0.161
**Social economic status** Low Middle High Missing	84 (30.9) 145 (53.3) 39 (14.3) 4 (1.5)	301 (33.5) 399 (44.4) 186 (20.7) 13 (1.4)	0.039
**Medical insurance** “Extra” insurance **vs. basic**	248 (91.2)	811 (90.2)	0.635
**Primary cancer site** Breast Gynecological cancers Gastrointestinal Lung Prostate Prostate (in men) Urinary Other	75 (27.6) 46 (16.9) 81 (29.8) 42 (15.4) 5 (1.8) 5 (5.7) 11 (4.0) 10 (3.7)	482 (53.6) 135 (15.0) 158 (17.6) 83 (9.2) 11 (1.2) 11 (6.9) 13 (1.3) 11 (1.2)	<0.001 0.449 <0.001 0.004 0.543 0.705 0.008 0.015
**Cancer recurrence** Yes	61 (22.6)	165 (18.5)	0.132
**Cancer metastasis** Yes	136 (51.3)	323 (36.9)	<0.001
**Oncology treatment setting** Adjuvant Neoadjuvant Palliative Curative	48 (18.8) 73 (28.5) 135 (52.7) 0	274 (32.0) 280 (32.7) 301 (35.1) 2 (0.2)	<0.001
**Comorbidity** Hypertension Hyperlipidemia Obesity Ischemic heart disease Chronic renal failure Cerebrovascular accident	203 (74.6) 246 (90.4) 137 (50.4) 56 (20.6) 31 (11.4) 21 (7.7)	288 (32.0) 488 (54.3) 206 (22.9) 55 (6.1) 28 (3.1) 24 (2.7)	<0.001 <0.001 <0.001 <0.001 <0.001 <0.001
Charlson Comorbidity Index Mean ± std Median (IQR)	5.93 ± 2.1 6 (5;7)	3.36 ± 1.9 3 (2;5)	<0.001
**Prior CM use** (non-cancer-related)	164 (60.3)	568 (63.2)	0.432
**Cancer-related CM use**	82 (30.1)	372 (41.4)	0.001

**Table 2:** Defined treatment goals in patients with diabetes mellitus (DM) compared to patients without DM. GI: gastrointestinal.

	All Patients			Breast			Gastrointestinal			Gynecological		
	DM n = 272	Non-DM n = 899	*p*	DM n = 75	Non-DM n = 482	*p*	DM n = 81	Non-DM n = 158	*p*	DM n = 46	Non-DM n = 135	*p*
Pain	166 (61)	523 (58.2)	0.229	46 (61.3)	270 (56)	0.149	46 (56.8)	88 (55.7)	0.454	37 (80.4)	95 (70.4)	0.219
Emotional concerns	178 (65.4)	605 (67.3)	0.828	42 (56)	341 (70.7)	0.038	50 (61.7)	104 (65.8)	0.649	37 (80.4)	85 (62.9)	0.034
GI concerns	204 (75)	663 (73.7)	0.408	51 (68)	353 (73.2)	0.673	65 (80.2)	123 (77.8)	0.468	40 (88.9)	97 (71.8)	0.045
Fatigue	213 (78.3)	713 (79.3)	0.851	55 (73.3)	387 (80.3)	0.577	66 (81.5)	123 (77.8)	0.333	36 (78.2)	103 (76.3)	0.912

**Table 3:** Quality of life (QoL) in patients with diabetes mellitus (DM) compared to that in patients without DM.

	All Patients			Breast			Gastrointestinal			Gynecological		
	DM n = 272	Non-DM n = 899	*p*	DM n = 75	Non-DM n = 482	*p*	DM n = 81	Non-DM n = 158	*p*	DM n = 46	Non-DM n = 135	*p*
^¥^ **ESAS**												
Pain	4.9 ± 3.4 5 (1;8)	4.4 ± 3.2 5 (2;7)	0.022	4.9 ± 3.3 5 (2;8)	4.2 ± 3.1 4 (1;7)	0.103	4.6 ± 3.5 5 (1;8)	4.1 ± 3.3 4 (1;7)	0.317	6.4 ± 2.7 7 (5;8)	4.9 ± 3.0 5 (2;8)	0.03
Fatigue	6.0 ± 2.9 7 (4;8)	5.8 ± 2.8 6 (4;8)	0.212	6.1 ± 3.2 7 (4;9)	5.8 ± 2.9 6 (3;8)	0.432	5.7 ± 3.0 6 (4;8)	5.9 ± 2.8 6 (4;8)	0.730	6.7 ± 2.6 7 (5;8)	5.9 ± 2.6 7 (4;8)	0.070
Nausea	2.2 ± 3.0 0 (0;4)	2.6 ± 3.1 1 (0;5)	0.043	2.2 ± 3.3 0 (0;4)	2.7 ± 3.2 1 (0;5)	0.057	2.7 ± 2.9 2 (0;6)	2.9 ± 3.2 2 (0;5)	0.766	2.2 ± 3.1 0 (0;4)	2.4 ± 2.7 1 (0;5)	0.508
Depression	3.9 ± 3.6 3.5 (0;7)	3.5 ± 3.2 3 (0;6)	0.112	3.9 ± 3.9 3 (0;8)	3.5 ± 3.2 3 (0;6)	0.805	3.3 ± 3.1 3 (0;6)	3.4 ± 3.2 3 (0;6)	0.883	4.7 ± 3.5 5 (1;8)	3.5 ± 3.4 3 (0;6)	0.052
Anxiety	4.2 ± 3.7 3.5 (0;8)	4.1 ± 3.4 4 (0.75;7)	0.979	4.1 ± 4.0 3 (0;9)	4.3 ± 3.4 4 (1;7)	0.540	3.6 ± 3.2 3 (0;6)	3.9 ± 3.4 3 (0;6)	0.632	5.1 ± 3.7 6 (1;9)	4.1 ± 3.5 4 (0;7)	0.126
Drowsiness	5.1 ± 3.3 5 (2;8)	5.0 ± 3.1 5 (2;8)	0.458	5.6 ± 3.4 6 (2;8)	5.1 ± 3.1 5 (3;8)	0.158	5.0 ± 3.1 5 (3;8)	5.1 ± 3.0 5 (3;8)	0.810	5.4 ± 3.3 6 (3;8)	4.5 ± 3.3 4 (2;7)	0.110
Breathing	2.2 ± 2.9 0 (0;4)	2.1 ± 2.9 0 (0;4)	0.754	2.0 ± 2.8 0 (0;3)	2.0 ± 2.8 0 (0;4)	0.917	1.8 ± 2.4 0 (0;4)	1.2 ± 2.4 0 (0;1)	0.032	2.6 ± 2.8 1 (0;5)	2.4 ± 3.1 1 (0;4)	0.921
Appetite	4.7 ± 3.5 5 (1;8)	4.4 ± 3.3 5 (1;7)	0.287	4.5 ± 3.5 5 (1;7)	4.2 ± 3.3 4 (0;7)	0.500	5.0 ± 3.5 5 (1;8)	4.9 ± 3.5 5 (2;8)	0.874	5.3 ± 3.3 5 (3;9)	4.6 ± 3.1 5 (2;7)	0.203
Sleep	4.7 ± 3.3 5 (2;8)	4.9 ± 3.2 5 (2;8)	0.494	4.7 ± 3.3 5 (1;8)	4.8 ± 3.2 5 (2;8)	0.742	4.3 ± 3.1 4 (2;7)	4.5 ± 3.0 5 (2;7)	0.578	5.5 ± 3.4 6 (2;8)	4.9 ± 3.2 5 (2;7)	0.216
Well-being	5.9 ± 2.7 6 (4;8)	5.5 ± 2.7 5 (4;8)	0.021	5.8 ± 2.8 6 (4;8)	5.5 ± 2.7 5 (3;8)	0.340	6.1 ± 2.7 6 (5;8)	5.5 ± 2.8 5 (3;7)	0.062	6.5 ± 2.6 7 (5;8)	5.7 ± 2.4 5 (4;8)	0.066
**^#^** **EORTC QLQ-C30:**												
Emotional functioning	53.6 ± 32.0 5 (33;83)	52.7 ± 30.6 58 (33;75)	0.626	54.8 ± 35.8 50 (25;92)	51.7 ± 30.4 50 (29;75)	0.469	54.4 ± 29.4 50 (33;75)	56.3 ± 31.0 58 (33;83)	0.678	43.7 ± 32.4 42 (8;75)	48.8 ± 31.4 50 (2575)	0.427
Pain	57.4 ± 36.2 67 (33;100)	52.7 ± 35.5 50 (17;83)	0.069	61.5 ± 33.3 67 (33;100)	50.8 ± 35.0 50 (17;83)	0.020	50 ± 34.4 50 (17;83)	48.3 ± 37.2 50 (17;83)	0.769	70.5 ± 32.3 83 (50;100)	58.5 ± 35.0 67 (33;92)	0.049
Fatigue	72.8 ± 28.1 78 (56;100)	71.7 ± 27.5 78 (56;100)	0.430	75.9 ± 26.7 89 (56;100)	71.6 ± 27.9 78 (56;100)	0.232	69.6 ± 28.6 78 (44;100)	69.3 ± 28.8 67 (56;100)	0.963	80.3 ± 25.5 89 (67;100)	73.1 ± 26.4 89 (67;100)	0.072
Nausea	19.8 ± 24.7 17 (0;33)	21.0 ± 23.9 17 (0;33)	0.281	22.8 ± 26.1 17 (0;33)	20.7 ± 23.2 17 (0;33)	0.708	24.0 ± 26.0 17 (0;33)	23.1 ± 23.7 17 (0;100)	0.953	15.8 ± 24.8 0 (0;17)	23.2 ± 26.6 17 (0;33)	0.053
Appetite	52.5 ± 39.8 67 (0;100)	48.4 ± 37.7 33 (0;66.7)	0.147	51.6 ± 39.4 67 (0;100)	45.7 ± 37.9 33 (0;67)	0.260	55.4 ± 38.5 67 (33;100)	55.1 ± 38.0 67 (33;100)	0.956	57.3 ± 39.0 67 (33;100)	47.3 ± 36.1 33 (25;67)	0.142
Sleep	51.3 ± 39.5 67 (0;100)	55.6 ± 37.2 67 (33;100)	0.144	52.3 ± 40.4 67 (0;100)	54.2 ± 37.3 67 (33;100)	0.777	50 ± 38.0 67 (0;67)	47.0 ± 36.7 33 (0;67)	0.583	53.5 ± 42.8 67 (0;100)	61.7 ± 35.8 67 (33;100)	0.356
Well-being	37.7 ± 22.8 42 (17;50)	38.2 ± 23.8 42 (17;5)	0.810	38.6 ± 23.5 42 (17;50)	39.6 ± 24.0 42 (17;58)	0.692	38 (17;50)	37.7 ± 23.7 33 (17;50)	0.637	34.0 ± 19.5 33 (17;50)	36.0 ± 22.9 33 (17;50)	0.711

^¥^ESAS: The Edmonton Symptom Assessment Scale is a scale from 0 (the lowest severity) to 10 (the greatest severity). ^#^EORTC QLQ-C30: European Organization for Research and Treatment of Cancer Quality of Life Questionnaire. Functioning scales have scores ranging from 0 (the greatest severity) to 100 (the lowest severity), while the symptom scales range from 0 (the lowest severity) to 100 (the greatest severity).

**Table 4:** A multivariate analysis for the association between DM and ESAS scores > 5 in different cancer types.

	Pain		Fatigue		Well-Being	
	OR 95% CI	*p*	OR 95% CI	*p*	OR 95% CI	*p*
**Diabetes**						
No	**Reference**		**Reference**		**Reference**	
Yes	1.47 (1.02–2.12)	0.038	0.79 (0.55–1.15)	0.223	1.26 (0.88–1.81)	0.209
**Cancer type**						
Breast	**Reference**		**Reference**		**Reference**	
Gynecological	1.77 (1.24–2.52)	0.002	1.27 (0.90–1.81)	0.179	1.04 (0.73–1.48)	0.842
Gastrointestinal	1.10 (0.73–1.66)	0.643	0.86 (0.62–1.19)	0.37	0.99 (0.72–1.38)	0.971

## Data Availability

The research data can be made available upon reasonable request by the corresponding author.

## Abbreviations

The following abbreviations are used in this manuscript:

QoL	Quality of life
DM	Diabetes mellitus
IO	Integrative oncology
ESAS	Edmonton Symptom Assessment Scale
EORTC	European Organization for Research and Treatment of Cancer
SIO	Society for Integrative Oncology
ASCO	American Society of Clinical Oncology

## References

[1] Suh S., Kim K.W. (2019). Diabetes and cancer: Cancer should be screened in routine diabetes assessment. Diabetes Metab. J..

[2] Barone B.B., Yeh H.-C., Snyder C.F., Peairs K.S., Stein K.B., Derr R.L., Wolff A.C., Brancati F.L. (2008). Long-term all-cause mortality in cancer patients with preexisting diabetes mellitus: A systematic review and meta-analysis. JAMA.

[3] Garg S.K., Maurer H., Reed K., Selagamsetty R. (2013). Diabetes and cancer: Two diseases with obesity as a common risk factor. Diabetes Obes. Metab..

[4] Joharatnam-Hogan N., Carter T.J., Reynolds N., Ho J.H., Adam S., Board R., Feingold K.R., Anawalt B., Boyce A., Chrousos G., de Herder W.W., Dhatariya K., Dungan K., Hershman J.M., Hofland J., Kalra S. (2000). Diabetes Mellitus in People with Cancer. Endotext.

[5] Peairs K.S., Barone B.B., Snyder C.F., Yeh H.-C., Stein K.B., Derr R.L., Brancati F.L., Wolff A.C. (2011). Diabetes mellitus and breast cancer outcomes: A systematic review and meta-analysis. J. Clin. Oncol..

[6] Sabatino S.A., Thompson T.D., Wu X.-C., Fleming S.T., Kimmick G.G., Trentham-Dietz A., Cress R., Anderson R.T. (2014). The influence of diabetes severity on receipt of guideline-concordant treatment for breast cancer. Breast Cancer Res. Treat..

[7] Vissers P.A.J., Falzon L., van de Poll-Franse L.V., Pouwer F., Thong M.S.Y. (2016). The impact of having both cancer and diabetes on patient-reported outcomes: A systematic review and directions for future research. J. Cancer Surviv..

[8] Mailliez A., Ternynck C., Duhamel A., Mailliez A., Ploquin A., Desauw C., Lemaitre M., Bertrand N., Vambergue A., Turpin A. (2023). Diabetes is associated with high risk of severe adverse events during chemotherapy for cancer patients: A single-center study. Int. J. Cancer.

[9] Kleckner A.S., Kleckner I.R., Culakova E., Shayne M., Belcher E.K., Gudina A.T., Williams A.M., Onitilo A.A., Hopkins J.O., Gross H. (2022). The association between cancer-related fatigue and diabetes from pre-chemotherapy to 6 months post-chemotherapy. Support. Care Cancer.

[10] Lavdaniti M., Michalopoulou S., Owens D.-A., Vlachou E., Kazakos K. (2021). The impact of comorbid diabetes type 2 on quality of life in cancer patients undergoing chemotherapy. Endocr. Metab. Immune Disord. Drug Targets.

[11] Phillips A.L., Reeves D.J., Storey S. (2023). Impact of diabetes (type 2) and glycemic control on health-related outcomes of patients receiving chemotherapy for non-metastatic breast cancer: A retrospective analysis. Support. Care Cancer.

[12] Kuo H.J., Chang N.T., Tien Y.W., Chou Y.J., Shun S.C. (2021). Determinants of Quality of Life in Individuals with a Dual Diagnosis of Resectable Pancreatic Cancer and Diabetes Mellitus. Oncol. Nurs. Forum..

[13] Ose D.J., Adediran E., Mark B., Ocier K., Dunson W.A., Turner C., Taylor B., Svoboda K., Post A.R., Leiser J. (2024). The association of diabetes mellitus and routinely collected patient-reported outcomes in patients with cancer. A real-world cohort study. Cancer Med..

[14] Choi K.-H., Park S.M., Lee K., Kim K.H., Park J.-S., Han S.H. (2013). Prevalence, awareness, control, and treatment of hypertension and diabetes in korean cancer survivors: A cross-sectional analysis of the fourth and fifth Korea national health and nutrition examination surveys. Asian Pac. J. Cancer Prev..

[15] Gilchrist S.C., Barac A., Ades P.A., Alfano C.M., Franklin B.A., Jones L.W., La Gerche A., Ligibel J.A., Lopez G., Madan K. (2019). Cardio-Oncology Rehabilitation to Manage Cardiovascular Outcomes in Cancer Patients and Survivors: A Scientific Statement from the American Heart Association. Circulation.

[16] Mao J.J., Pillai G.G., Andrade C.J., Ligibel J.A., Basu P., Cohen L., Khan I.A., Mustian K.M., Puthiyedath R., Dhiman K.S. (2022). Integrative oncology: Addressing the global challenges of cancer prevention and treatment. CA Cancer J. Clin..

[17] Mao J.J., Ismaila N., Bao T., Barton D., Ben-Arye E., Garland E.L., Greenlee H., Leblanc T., Lee R.T., Lopez A.M. (2022). Integrative Medicine for Pain Management in Oncology: Society for Integrative Oncology-ASCO Guideline. J. Clin. Oncol..

[18] Carlson L.E., Ismaila N., Addington E.L., Asher G.N., Atreya C., Balneaves L.G., Bradt J., Fuller-Shavel N., Goodman J., Hoffman C.J. (2023). Integrative Oncology Care of Symptoms of Anxiety and Depression in Adults with Cancer: Society for Integrative Oncology-ASCO Guideline. J. Clin. Oncol..

[19] Bower J.E., Lacchetti C., Alici Y., Barton D.L., Bruner D., Canin B.E., Escalante C.P., Ganz P.A., Garland S.N., Gupta S. (2024). Management of Fatigue in Adult Survivors of Cancer: ASCO-Society for Integrative Oncology Guideline Update. J. Clin. Oncol..

[20] Ben-Arye E., Hausner D., Samuels N., Gamus D., Lavie O., Tadmor T., Gressel O., Agbarya A., Attias S., David A. (2022). Impact of acupuncture and integrative therapies on chemotherapy-induced peripheral neuropathy: A multicentered, randomized controlled trial. Cancer.

[21] Ben-Arye E., Elly D., Samuels N., Gressel O., Shulman K., Schiff E., Lavie O., Minerbi A. (2021). Effects of a patient-tailored integrative oncology intervention in the relief of pain in palliative and supportive cancer care. J. Cancer Res. Clin. Oncol..

[22] Zhao Y., Yue R. (2024). Aging adipose tissue, insulin resistance, and type 2 diabetes. Biogerontology.

[23] Berkowitz S.A., Fabreau G.E., Raghavan S., Kentoffio K., Chang Y., He W., Atlas S.J., Percac-Lima S. (2016). Risk of developing diabetes among refugees and immigrants: A longitudinal analysis. J. Community Health.

[24] Volaco A., Cavalcanti A.M., Filho R.P., Précoma D.B. (2018). Socioeconomic status: The missing link between obesity and diabetes mellitus?. Curr. Diabetes Rev..

[25] Formica V., Tesauro M., Cardillo C., Roselli M. (2012). Insulinemia and the risk of breast cancer and its relapse. Diabetes Obes. Metab..

[26] American Diabetes Association Professional Practice Committee (2025). 4. Comprehensive Medical Evaluation and Assessment of Comorbidities: Standards of Care in Diabetes-2025. Diabetes Care.

[27] Jensen T.S., Karlsson P., Gylfadottir S.S., Andersen S.T., Bennett D.L., Tankisi H., Finnerup N.B., Terkelsen A.J., Khan K., Themistocleous A.C. (2021). Painful and non-painful diabetic neuropathy, diagnostic challenges and implications for future management. Brain.

[28] Ewertz M., Qvortrup C., Eckhoff L. (2015). Chemotherapy-induced peripheral neuropathy in patients treated with taxanes and platinum derivatives. Acta Oncol..

[29] Trendowski M.R., Lusk C.M., Ruterbusch J.J., Seaton R., Simon M.S., Greenwald M.K., Harper F.W.K., Beebe-Dimmer J.L., Schwartz A.G. (2021). Chemotherapy-induced peripheral neuropathy in African American cancer survivors: Risk factors and quality of life outcomes. Cancer Med..

[30] Xie Y., Wang Q., Hu T., Chen R., Wang J., Chang H., Cheng J. (2021). Risk Factors Related to Acute Radiation Dermatitis in Breast Cancer Patients After Radiotherapy: A Systematic Review and Meta-Analysis. Front Oncol..

[31] Ben-Arye E., Lopez A.M., Daoud N., Zoller L., Walker E., Davidescu M., Shulman K., Gressel O., Stein N., Brosh S. (2024). Identifying factors associated with disparities in access to integrative oncology program. J. Pain Symptom Manag..

